# Inheritance of pre-emergent metribuzin tolerance and putative gene discovery through high-throughput SNP array in wheat (*Triticum aestivum* L.)

**DOI:** 10.1186/s12870-019-2070-x

**Published:** 2019-10-29

**Authors:** Roopali Bhoite, Ping Si, Hui Liu, Ling Xu, Kadambot H. M. Siddique, Guijun Yan

**Affiliations:** 1 UWA School of Agriculture and Environment, The University of Western Australia, Perth, WA 6009 Australia; 2 The UWA Institute of Agriculture, The University of Western Australia, Perth, WA 6009 Australia; 3 Zhejiang Province Key Laboratory of Plant Secondary Metabolism and Regulation, College of Life Sciences, Zhejiang Sci-Tech University, Hangzhou, 310018 People’s Republic of China

**Keywords:** Metribuzin, Gene effects, Inheritance, Potence ratio, Heritability, Wheat 90 K iSelect SNP genotyping assay, Candidate genes

## Abstract

**Background:**

Herbicide tolerance is an important trait that allows effective weed management in wheat crops in dryland farming. Genetic knowledge of metribuzin tolerance in wheat is needed to develop new cultivars for the industry. Here, we investigated gene effects for metribuzin tolerance in nine crosses of wheat by partitioning the means and variances of six basic generations from each cross into their genetic components to assess the gene action governing the inheritance of this trait. Metribuzin tolerance was measured by a visual senescence score 21 days after treatment. The wheat 90 K iSelect SNP genotyping assay was used to identify the distribution of alleles at SNP sites in tolerant and susceptible groups.

**Results:**

The scaling and joint-scaling tests indicated that the inheritance of metribuzin tolerance in wheat was adequately described by the additive-dominance model, with additive gene action the most significant factor for tolerance. The potence ratio for all the crosses ranged between − 1 and + 1 for senescence under metribuzin-treated conditions indicating a semi-dominant gene action in the inheritance of metribuzin tolerance in wheat. The number of segregating genes governing metribuzin tolerance was estimated between 3 and 15. The consistent high heritability range (0.82 to 0.92) in F_5–7_ generations of Chuan Mai 25 (tolerant) × Ritchie (susceptible) cross indicated a significant contribution of additive genetic effects to metribuzin tolerance in wheat. Several genes related to photosynthesis (e.g. photosynthesis system II assembly factor YCF48), metabolic detoxification of xenobiotics and cell growth and development (cytochrome P450, glutathione S-transferase, glycosyltransferase, ATP-binding cassette transporters and glutathione peroxidase) were identified on different chromosomes (2A, 2D, 3B, 4A, 4B, 7A, 7B, 7D) governing metribuzin tolerance.

**Conclusions:**

The simple additive–dominance gene effects for metribuzin tolerance will help breeders to select tolerant lines in early generations and the identified genes may guide the development of functional markers for metribuzin tolerance.

## Background

Wheat (*Triticum aestivum* L.) is a major global cereal crop in terms of production and area coverage (FAO 2018) [1]. Wheat is Australia’s largest grain crop and contributes around 12% of world trade. Western Australia (WA) has the highest reported occurrence of herbicide-resistant weeds in Australia, which is the key agronomic issue for WA farmers. There are instances where weed infestations have caused serious reductions (up to 50%) in wheat yields [2]. Higher tolerance for metribuzin is advantageous for WA wheat industry to protect crops against herbicide damage and maximize crop yields. Therefore, breeding wheat cultivars for higher herbicide tolerance through improvement programs is paramount, particularly in Mediterranean-type climatic regions.

Inheritance of metribuzin tolerance has a different modes of genetic control in crop plants. A monogenic recessive inheritance was reported in soyabean (*Glycine max* L.) [3, 4] and potato (*Solanum tuberosum* L.) [5]. Si et al. [6] reported two independent semi-dominant genes having additive effects in narrow-leafed lupin (*Lupinus angustifolius* L.). The inheritance of tolerance to metribuzin in durum wheat (*T. turgidum* L.) is a complex character controlled by both nuclear and cytoplasmic genes in wheat [7, 8]. This was supported by the observation that physiological processes, such as uptake, translocation and metabolism/detoxification, modified the amount of herbicide reaching the target site. Investigations into the genetic control and heritability of metribuzin tolerance will guide breeders to formulate the appropriate selection program for the breeding of herbicide tolerant cultivars.

Variation in metribuzin tolerance in wheat from six continents, reported in our previous investigation [9], provides a valuable source to breeders for estimating gene effects and formulating advantageous breeding procedures to improve herbicide tolerance. The natural variability observed between genotypes for metribuzin tolerance indicates that selection may be an effective method for improving yields. However, selection efficiency is related to the magnitude of heritability and genetic advances. Heritability estimates along with genetic advances are important selection parameters, and usually more helpful for predicting genetic gain under selection [10]. Therefore, a detailed understanding of the nature of gene action, heritability and predicted genetic gain is helpful for selecting superior wheat germplasm in breeding programs to improve herbicide tolerance and yield.

DNA markers have enormous potential for improving the efficiency and precision of conventional plant breeding via marker-assisted selection (MAS). The molecular mechanism of metribuzin tolerance in wheat is poorly understood. Advances in next-generation sequencing have facilitated the discovery of SNPs in the whole genome [11, 12] to provide a large amount of genome-wide polymorphism, as they potentially represent all the mutations that have occurred in the genome [13, 14]. The recent wheat 90 K SNP iSelect assay developed by Illumina is a useful genetic resource for tagging agronomically important traits. The closed-end assay incorporates existing sequence knowledge onto a microarray platform enabling high-throughput SNP discovery in diverse pools.

This study aimed to (1) characterize the inheritance of tolerance to metribuzin in nine wheat crosses, (2) investigate heritability in F_5–7_ RILs of the most diverse cross (Chuan Mai 25 × Ritchie) (3) conduct 90 K iSelect SNP genotyping assay in diverse cultivars to discover allelic variants in SNP markers in tolerant and susceptible groups, and (4) determine the likely chromosomal locations and candidate genes responsible for metribuzin tolerance in wheat.

## Results

### Phenotypic variation

The average senescence (SS) for the tolerant and susceptible parents used in this study are in Table 1. The susceptible parents had significantly (*P* < 0.05) higher SS than the tolerant parents. Average SS for F_1_, F_2_, BC_T_, and BC_S_ populations are in Table 2. The ANOVA indicated a highly significant difference between generations, indicating genetic variability for metribuzin tolerance in wheat. F_2_ means had a comparable range to F_1_ means. The mean SS of the backcrosses varied depending on the crossed parents. The abbreviations representing crosses are in Table 1. Backcrossing F_1_ lines (BC_T_) to tolerant parents had lower SS than the mid-parent (mp) value, except for the K × D cross, indicating positive additive gene action and higher expression of metribuzin tolerance. In contrast, BC_S_ had higher SS than the mid-parent value. The crosses of F_1_ with susceptible Dagger differed the most from the mp value, by 31.3, 38.6 and 29% for the crosses CM × D, F × D and K × D, respectively. The comparisons of reciprocal crosses revealed significant differences (*P* ≤ 0.05) in average SS except for three reciprocal cross combinations (Table 3). Therefore the reciprocal crosses were not pooled for generation mean analysis.

**Table 1:** Origin and average senescence score of seven wheat genotypes used in crosses

Cultivar	Origin	Senescence score^a^	Reaction^b^
Chuan Mai 25 (CM)	China, Asia	3.05 ± 0.47	T
Dagger (D)	Australia	7.90 ± 0.25	S
Eagle Rock (ER)	Australia	3.95 ± 0.17	MT
Fundulea 490 (F)	Romania, Europe	4.40 ± 0.27	MT
Kite (K)	Australia	3.20 ± 0.34	T
Ritchie (R)	Europe	7.80 ± 0.32	S
Spear (S)	Australia	6.40 ± 0.11	S

^*a*^ Pre-emergent metribuzin rate of 400 g ai ha^− 1^ was sprayed and phytotoxicity was measured in wheat seedlings, 21 DAT. See text for details about senescence scaling. Data represented are mean and standard error

^*b*^ Cultivar reaction to metribuzin; T tolerant; MT moderately tolerant; S susceptible

**Table 2:** Generation means of senescence score (standard error in parenthesis) and potence ratio showing relationship between alleles for reaction to metribuzin in wheat crosses

Cross (♀ × ♂)^γ^	Senescence score means					Potence ratio
	MP	F1	F2	BC_T_	BC_S_	
CM × R	5.42	3.64 (0.2)^b^	4.45 (0.16)^b^ (285)^c^	3.35 (0.15)^b^ (16)^c^	6.77 (0.20)^b^ (11)^c^	− 0.75
CM × S	4.72	4.8 (0.2)^b^	3.17 (0.11)^b^ (343)^c^	3.78 (0.11)^b^ (16)^c^	5.2 (0.11)^b^ (10)^c^	0.04
CM × D	5.48	5.86 (0.4)^b^	5.4 (0.14)^b^ (428)^c^	5.0 (0.10)^b^ (17)^c^	7.20 (0.12)^b^ (12)^c^	0.16
ER × R	5.87	4.8 (0.37)^b^	6.27 (0.09)^b^ (692)^c^	5.23 (0.15)^b^ (13)^c^	6.41 (0.13)^b^ (12)^c^	− 0.56
ER × S	5.17	5.8 (0.32)^b^	6.24 (0.15)^b^ (356)^c^	4.09 (0.13)^b^ (12)^c^	5.68 (0.13)^b^ (11)^c^	0.51
ER × D	5.92	6.8 (0.42)^b^	5.99 (0.12)^b^ (412)^c^	5.41 (0.13)^b^ (12)^c^	6.96 (0.17)^b^ (12)^c^	0.44
F × R	6.10	6.5 (0.37)^b^	–^a^	–^a^	–^a^	0.24
F × S	6.10	5.86 (0.79)^b^	5.02 (0.11)^b^ (610)^c^	5.68 (0.11)^b^ (16)^c^	6.54 (0.11)^b^ (12)^c^	0.46
F × D	6.15	6.7 (0.25)^b^	6.55 (0.12)^b^ (476)^c^	6.91 (0.19)^b^ (17)^c^	8.53 (0.15)^b^ (13)^c^	0.31
K × S	4.80	4.2 (0.58)^b^	–^a^	–^a^	–^a^	−0.38
K × D	5.55	5.25 (0.41)^b^	5.55 (0.11)^b^ (475)^c^	6.03 (0.18)^b^ (13)^c^	7.15 (0.15)^b^ (10)^c^	−0.13

*MP* = mid-parental value, calculated as (P_1_ + P_2_)/2, *BC*_*T*_ and *BC*_*S*_ represent backcross of F_1_ to tolerant and susceptible parents, respectively

^*a*^ Data not available

^*b*^ Standard error

^*c*^ Number of RILs

^γ^ Abbreviated cultivar names based on Table 1

**Table 3:** Observed metribuzin tolerance measured as senescence score in reciprocal crosses following application of metribuzin (400 g a.i. ha^− 1^)

Crosses^γ^	F_1(F)_^a^	F_1(M)_^b^	*P* value
CM × R	3.64 (0.2)^d^ (11)^e^	–^c^	– ^c^
CM × S	4.8 (0.2)^d^ (7)^e^	–^c^	–^c^
CM × D	5.86 (0.4)^d^ (7)^e^	9 (0.32)^d^ (8)^e^	0.00**
ER × R	4.8 (0.37)^d^ (8)^e^	7.4 (0.24)^d^ (7)^e^	0.00**
ER × S	5.8 (0.32)^d^ (10)^e^	3.8 (0.86)^d^ (6)^e^	0.05*
ER × D	6.8 (0.42)^d^ (9)^e^	7.27 (0.48)^d^ (11)^e^	0.03*
F × R	6.5 (0.37)^d^ (8)^e^	–^c^	– ^c^
F × S	5.86 (0.79)^d^ (7)^e^	6 (0.44)^d^ (6)^e^	1 (NS)
F × D	6.7 (0.25)^d^ (8)^e^	7.5 (0.56)^d^ (6)^e^	0.23 (NS)
K × R	–^c^	3.71 (0.18)^d^ (7)^e^	– ^c^
K × S	4.2 (0.58)^d^ (6)^e^	5.14 (0.76)^d^ (7)^e^	0.48 (NS)
K × D	5.25 (0.41)^d^ (8)^e^	4.90 (0.56)^d^ (10)^e^	0.04*

^*a*^ Mean value of the F1 derived from the line as tolerant female crossed with susceptible male

^*b*^ Mean value of the F1 derived from the line as susceptible female crossed with tolerant male

^*c*^ Data not available

^*d*^ Standard error

^*e*^ Number of RILs

^γ^ Abbreviated cultivar names based on Table 1

**, significant at *P* < 0.01; *, significant at *P* < 0.05; *NS,* not significant

### Genetic effects

#### Genetic model and gene action of metribuzin tolerance in wheat

The results of the scaling tests (A, B, C and D) of nine hybrids (Table 4) were not significant, which indicated the absence of epistatic gene interaction and adequacy of the simple additive–dominance model. The genetic parameters for mp, additive gene effects (d) and dominance gene effects (h) and their standard deviations estimated by the joint–scaling test are presented in Table 4. The mp, which reflects the contribution of the locus effects and interaction of fixed loci, were significant for all nine crosses. The additive gene effects were significant (*P* = 0.05) for all nine crosses, and dominance gene effects were significant (*P* = 0.05) for four crosses (CM × R, CM × S, F × D and ER × D). The additive-dominance model fitted well for all crosses. The model significance was checked using χ^2^ statistic, which showed insignificant difference between the expected and observed generation mean values, confirming a significant additive–dominance model for metribuzin tolerance in wheat (Table 4).

**Table 4:** Genetic model testing based on A, B, C and D scales and estimates of additive and dominance effects (standard error in parenthesis) for metribuzin tolerance in wheat

Cross (♀ × ♂)^γ^	Scales				Gene effects			χ2^a^
	A	B	C	D	Mean (m)	Additive effect (d)	Dominance effect (h)	
CM × R	0.01 (2.54)	2.10 (2.14)	−0.33 (10.92)	− 1.22 (5.32)	5.54 (0.20)**	2.78 (0.18)**	−1.67 (0.36)**	1.21 (NS)
CM × S	−0.29 (2.34)	−0.80 (0.96)	−6.37 (8.96)	− 2.64 (4.36)	4.88 (0.15)**	1.49 (0.13)**	−1.66 (0.34)**	7.43 (NS)
CM × D	1.09 (2.48)	0.64 (1.78)	−1.07 (10.18)	−1.40 (4.86)	5.72 (0.19)**	2.18 (0.13)**	0.37 (0.38) (NS)	0.58 (NS)
ER × R	1.71 (1.61)	0.22 (1.90)	3.73 (10.55)	0.90 (5.19)	5.92 (0.15)**	1.65 (0.13)**	0.09 (0.31) (NS)	2.98 (NS)
ER × S	−1.57 (1.60)	−0.84 (1.46)	3.01 (11.80)	2.71 (5.83)	5.10 (0.09)**	1.32 (0.08)**	0.36 (0.22) (NS)	1.81 (NS)
ER × D	0.07 (1.75)	−0.78 (2.19)	−1.49 (9.86)	−0.39 (4.70)	5.82 (0.14)**	1.80 (0.12)**	0.64 (0.29)**	0.00 (NS)
F × S	1.10 (2.58)	0.82 (2.30)	2.44 (12.56)	− 2.18 (5.90)	5.45 (0.12)**	0.93 (0.10)**	0.58 (0.27) (NS)	1.64 (NS)
F × D	2.72 (2.13)	2.46 (1.72)	0.50 (11.32)	−2.34 (5.64)	6.56 (0.15)**	1.73 (0.13)**	0.9 (0.29)*	3.70 (NS)
K × D	3.61 (2.31)	1.15 (1.86)	0.60 (9.11)	−2.08 (4.38)	5.88 (0.18)**	1.86 (0.15)**	0.14 (0.37) (NS)	3.48 (NS)

*A, B, C, D,* Scaling tests; χ2, Significance of the joint scaling test determined by the χ^2^test and observed and expected ‘t’ values compared at 5 and 1% level of significance

** Indicates significant difference at *P* ≤ 0.01; * Indicates significant difference at *P* ≤ 0.01

*NS,* not significant

^γ^ Abbreviated cultivar names based on Table 1

**Fig. 1:**
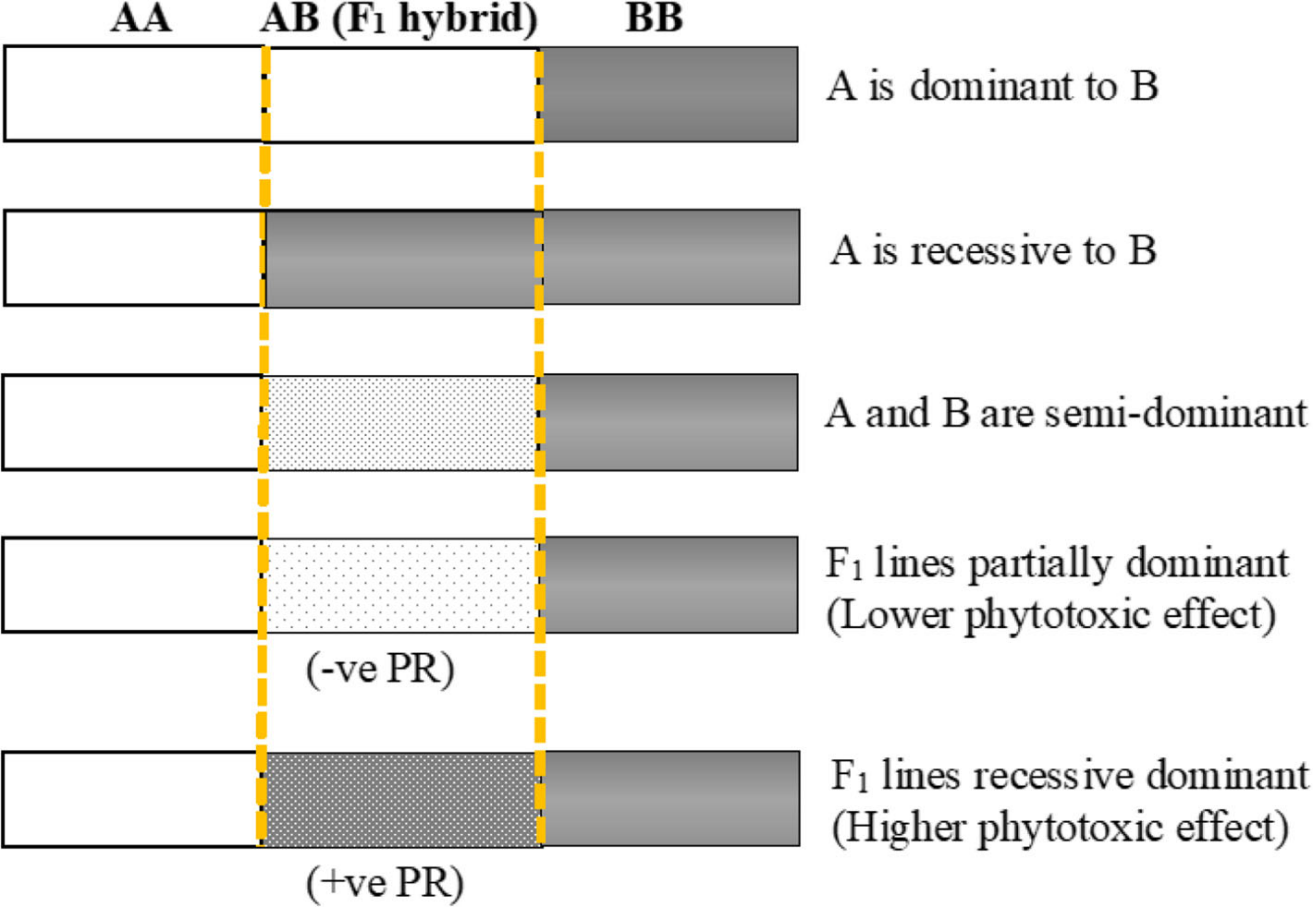
Dominance relationships between a pair of alleles A and B. Phenotypes corresponding to the different genotypes AA, AB and BB. -*ve PR*, negative potence ratio; F1 mean phenotypic value is similar to tolerant phenotypic value +*ve PR*, positive potence ratio; F1 mean phenotypic value is similar to susceptible phenotypic value

Metribuzin tolerance in wheat is either partially dominant or recessive dominant (Fig. 1). The potence ratio presented in Table 2 ranged from − 0.75 to 0.51 for SS under metribuzin– treated conditions, thereby falling between − 1 and + 1, indicating a semi-dominant gene action for the inheritance of metribuzin tolerance in wheat. The crosses with a negative potence ratio (CM × R, F × S, ER × R, K × S and K × D) had lower F_1_ means (lower phytotoxic effect) and were more similar to the tolerant parents, indicating the presence of partial dominance gene effects. The crosses with a positive potence ratio (CM × S, CM × D, F × R, F × D, ER × S, ER × D) had higher F_1_ means (higher phytotoxic effect), indicating recessive dominance (Fig. 1).

### Heritability and the number of resistance genes

**Fig. 2:**
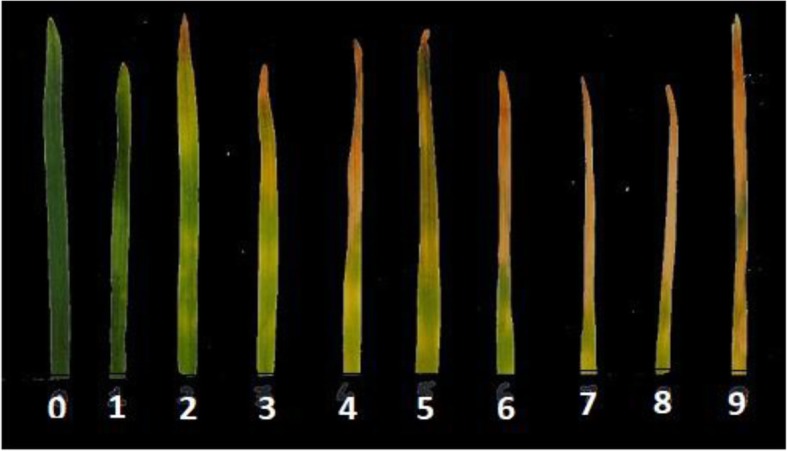
Phenotypic distribution of senescence score in Chuan Mai 25/Ritchie F5–-7 RIL population. *P*_*1*_ indicates the means of Chuan Mai 25 *P*_*2*_ indicates the means of Ritchie

The frequency distribution of the metribuzin reaction of F_5–7_ RILs of the Chuan Mai 25 × Ritchie appeared to be normal, indicating metribuzin tolerance as a quantitative trait (Fig. 2). The population means remained higher than those of the parents, indicating transgressive segregations in both directions of parents. Heritability was high and comparable in F_5_ (0.82), F_6_ (0.95) and F_7_ (0.92) RILs of the cross Chuan Mai 25/Ritchie (Table 5). There were minimum of eight major peaks representing major genes and some minor modifier genes in the F_5_, F_6_ and F_7_ RIL populations. Gene number, n_1_, estimated based on variances of parents and F_2_ and gene number n_2_, estimated based on variances of parents, F_1_ and F_2_ varied for most of the crosses. Wright’s formula estimated a minimum of three genes and a maximum of 15 genes controlling metribuzin tolerance in wheat (Table 6).

**Table 5:** Genotypic and phenotypic coefficients of variation, and broad sense heritability of metribuzin tolerance in wheat

Population	No. lines	Range of SS^a^	MSg	MSe	${\delta}_g^2$	${\delta}_e^2$	${\delta}_p^2$	H^2^
F_5_	73	1.7–10.0	32.29**	5.70	8.86	1.90	10.76	0.82
F_6_	73	2.0–10.0	110.44**	5.22	35.07	1.74	36.81	0.95
F_7_	73	1.7–10.0	60.73**	4.48	18.75	1.49	20.24	0.92

^*a*^ Minimum and maximum senescence score

*MSg* mean square of genotype; *MSe,* mean square of random error; ${\delta}_g^2,$ estimated genetic variance; ${\delta}_p^2,$ estimated phenotypic variance; ${\delta}_e^2,$ estimated error variance; *H*^*2*^*,* broad sense heritability

*F*_*5*_*, F*_*6*_*, F*_*7,*_ single-seed descent recombinant-inbred lines of, Chuan Mai 25 × Ritchie cross

** Indicates significant difference at *P* < 0.01

**Table 6:** Estimates of the minimum number of genes for metribuzin tolerance measured by senescence score

Cross^γ^	n_1_^a^	n_2_^b^	Mean
CM × R	11.94	15.14	13.54
CM × S	3.25	4.77	4.01
CM × D	8.76	11.29	10.02
ER × R	9.82	10.39	10.10
ER × S	5.99	5.75	5.87
ER × D	9.06	8.38	8.72
F × S	3.91	3.00	3.45
F × D	9.80	10.44	10.12
K × D	7.95	8.52	8.23

^*a*^ Minimum gene number, *n*_*1*_ = (P_1_–P_2_)^2^/8{V_F2_– [(V_P1_ + V_P2_)/2]}

^*b*^ Minimum gene number, *n*_*2*_ = (P_1_–P_2_)^2^/8{V_F2_– [(V_P1_ + V_P2_ + 2V_F1_)/4]}

^γ^ Abbreviated cultivar names based on Table 1

### SNP discovery and potential candidate genes

**Fig. 3:**
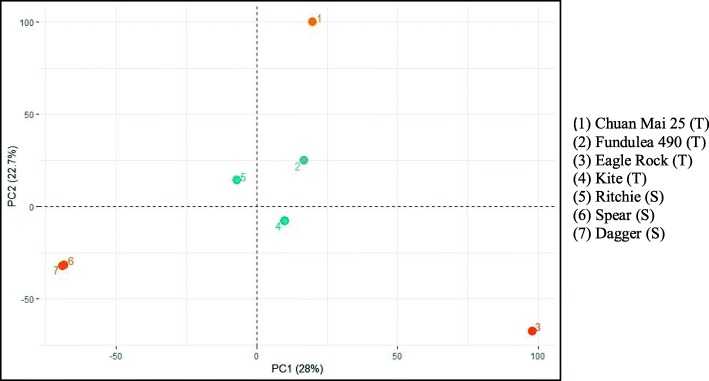
Principal component analysis showing genetic diversity based on 8,661 SNPs. Each point represents one individual. Principal component 1 (PC1) explains 28% of the variation and principal component 2 (PC2) explains 22.7% of the variation in the data

The 90 K iSelect SNP genotyping assay contained 81,587 SNPs. A total of 60,635 monomorphic alleles (74%) with no clustering patterns for all genotypes were removed. A total of 12,294 loci had no call and were removed. The remaining 8,661 loci (12.9%) had ≥2 clusters and were used for principal component analysis (PCA) analysis; the results for allelic variation in seven genotypes are presented in Fig. 3. The PCA analysis revealed significant variation between tolerant and susceptible groups. A clear separation of tolerant and susceptible groups, according to PCA component 1, indicated high genetic diversity between the two groups. A total of 296 SNPs were polymorphic/biallelic markers between the two groups (Additional file 1: Table S1).

Putative genes related to the identified SNPs with differences between tolerant and susceptible groups were investigated by a blastN search of markers on *Triticum aestivum* IWGSC_refseqv1.0. The results suggested that metribuzin tolerance was a quantitative trait governed by several loci on different chromosomes (2A, 2D, 3B, 4A, 4B, 7A, 7B, 7D) (Table 7). Only genes related to photosynthesis and metabolic detoxification pathways were considered for candidate gene analysis**.** Multiple SNPs and candidate genes identified on chromosome 7B (photosynthesis system II assembly factor YCF48 and ABC transporter), chromosome 4A (cytochrome P450 family), chromosome 7A (glutathione S-transferase), chromosome 2A and 3B (glycosyltransferase), and chromosome 2D (glutathione peroxidase) represented the possible genes/gene families with significant association with metribuzin tolerance in wheat.

**Table 7:** List of 12 candidate genes with a known function related to photosynthesis and metabolic detoxification

SNP name	Chromosome	A/B allele	Tolerant allele	Overlapping gene ID	Length (bp) and direction	Molecular function	Biological process
wsnp_Ex_c13505_21253168	3B	A/G	B	TRAES_3BF091600250CFD_c1	3614–	Glycosyltransferase activity	Sucrose synthase activity xenobiotics degradation
Kukri_c5295_1015	3B	T/G	A	TraesCS3B02G461800	3614–	Glycosyltransferase activity	Sucrose metabolic process xenobiotics degradation
BS00015680_51	2D	T/C	B	TraesCS2D02G598000	822–	Glutathione peroxidase (oxidoreductase, Peroxidase)	Protection from oxidative damage
Kukri_c2937_649	2A	A/G	B	TraesCS2A02G210100	3125–	Glycosyltransferase activity	Metabolic detoxification/ xenobiotics degradation
CAP11_c3631_75	4B	A/G	B	TraesCS4B02G056800	1564+	Kinase and transferase activity	ATP-, metal-, magnesium- and nucleotide-binding
BS00040929_51	7A	A/G	B	TraesCS7A02G130600	1495+	Glutathione S-transferase activity	Phase II metabolic isozymes involved in xenobiotic detoxification
Kukri_c1831_1243	4A	A/G	B	TraesCS4A02G446700	3084–	Sucrose synthase activity	Sucrose-cleaving enzyme that provides UDP-glucose and fructose for various metabolic pathways
tplb0060b03_921	7B	T/C	A	TraesCS7B02G486500	1549–	Photosynthesis system II assembly factor YCF48	YCF48 is necessary for efficient assembly and repair of the PSII.
RAC875_c16644_491	7D	A/G	A	TraesCS7D02G258300	1381+	Ubiquitination pathway	Stress response, DNA repair, signal transduction, cell-cycle control, transcriptional regulation and vesicular traffic.
Tdurum_contig10482_110	4A	T/C	A	TraesCS4A02G445600	1952+	Monooxygenase, oxidoreductase, iron and metal binding	Cytochrome P450 family metabolize potentially toxic compounds including drugs and products of endogenous metabolism
GENE-1887_85	3B	T/G	A	TraesCS3B02G045400	1437–	Oxidoreductase activity	Catalysis of oxidation-reduction reaction
Tdurum_contig14460_561	7B	T/C	A	TraesCS7B02G016400	3807–	ATP- and nucleotide-binding; hydrolysis of ATP to energize diverse biological systems.	ABC module is known to bind and hydrolyze ATP in numerous biological processes including multiple drug resistance

*Gene ID is the TRAES number according to the URGI-Jbrowse database on Ensembl Plants release; +/− indicates the direction (forward/reverse) on the strand; bp indicates base pairs*

## Discussion

The mode of inheritance and gene action of pre-emergent herbicide tolerance will help breeders to choose appropriate breeding methods to develop more tolerant cultivars and combat early weed competition to enhance wheat yields. The efficiency of selection and plant breeding programs depend on the existence of genetic variability [15]. Genetic variation for metribuzin tolerance in wheat was evident in our previous research [9, 16]. Metribuzin tolerance/sensitivity is controlled by both cytoplasmic and nuclear genes because reciprocal differences in expression of metribuzin tolerance existed in most F_1_ hybrids. Previously, Ratliff et al. [7] reported the role of both nuclear and cytoplasmic genes in metribuzin tolerance in wheat. Metribuzin tolerance is a polygenic trait and the present investigation revealed a maximum of 15 genes responsible for the trait. Villarroya et al. [8] reported metribuzin tolerance as a quantitative trait controlled by many genes in wheat, which supports the present findings. The Transgressive phenotypes observed in segregated populations (Fig. 2) compared to parental phenotypes were due to recombination of additive alleles both on positive and negative direction. Recombination results in new pairs of alleles at two or more loci. The changed/enhanced gene expression at these loci give rise to new phenotypes [17].

Metribuzin tolerance is explained by the simple additive–dominance model, indicating absence of epistasis or non-allelic interaction. The absence of epistasis and significant additive effect efficiently responds to selection [18]. The alleles of such traits are fixed in early generations. These facts can guide breeders in the selection of lines in early generations. The results of the scaling and joint-scaling tests and chi-square statistic can be used as evidence that the additive gene effect is higher than the dominance gene effect, indicating the former as a decisive type of gene action for metribuzin tolerance. Highly significant additive gene effects (d) for all crosses indicated the preponderance of additive gene effects for metribuzin tolerance and the potential for improving the performance of chlorophyll traits using early a pedigree selection program in wheat.

Dominance in genetics is a relationship between the alleles of one gene, where the effect on phenotype of one allele masks the contribution of a second allele at the same locus. It is a key concept in Mendelian inheritance and classical genetics. Often the dominant allele codes for a functional protein whereas the recessive allele does not [19]. In quantitative genetics, phenotypes are measured and treated numerically. In the present investigation, F_1_ hybrids with lower SS exhibited a partial dominant gene action. Therefore the F_1_ hybrids with a negative potence ratio had mid- to low- metribuzin phytotoxic effects and expressed a phenotype similar to the tolerant parent (Fig. 1). However, F_1_ hybrids with higher SS had recessive, dominant gene action. Therefore, the F_1_ hybrids with a positive potence ratio had mid- to high- metribuzin phytotoxic effects and expressed a phenotype similar to the susceptible parent.

Heritability was consistent and above 80% in the F_5–7_ RIL population of Chuan Mai 25 × Ritchie, which indicated stability of the metribuzin tolerance trait. These traits could be easily transferred through generations in breeding programs to generate more tolerant cultivar. The absence of epistasis increased the accuracy of the gene number estimate in the present study because it complied with Wright’s assumption of no epistasis [20]. The crosses had unidirectional distribution of genes based on the degree of susceptibility in susceptible parents. The crosses involving Ritchie as the susceptible parent segregated the most genes, followed by Dagger and Spear.

The candidate genes identified for SNPs having homozygous allele in the tolerant group encodes for the network of xenobiotic detoxification proteins protecting cells from oxidative damage and keeping the photosynthesis process intact by PSII complex repair under stress. The identified gene superfamilies or domains, notably cytochrome P450 (CYPs) and glutathione S-transferase (GSTs) glycosyltransferase (GT), ATP-binding cassette transporters and glutathione peroxidase (GPX) are essentially xenobiotic detoxifying enzymes involved in vacuolar sequestration of conjugated pesticide metabolites [21–23]. Plants can metabolize a diverse range of xenobiotics, such as organic pollutants and pesticides, and herbicides using enzymes [22]. The most commonly observed route for the detoxification of herbicides in wheat involves an initial hydroxylation, typically mediated by a cytochrome P450 mixed function oxidases (CYPs) and glutathione conjugation mediated by glutathione S-transferases (GSTs). CYPs and GSTs are implicated in metabolism-based resistance to multiple herbicides in grass weeds such as black-grass [24].

The identified glycosyltransferase and oxidoreductase mediate different biological processes. They are involved in sucrose metabolism and metabolic detoxification of xenobiotic detoxification. The candidate genes detected from our previous investigation [16] of QTL mapping suggested glycosyltransferase and oxidoreductase involved in metabolic detoxification, partially imparts metribuzin tolerance in wheat. The microarray analysis conducted by Pilcher et al. [25] revealed that sucrose metabolism was highly responsive to metribuzin stress in wheat. The identified photosystem (PS) II assembly factor YCF48 is the thylakoid-embedded large pigment-protein complexes of photosynthetic electron transfer chain, i.e. PSII, PSI, the cytochrome b_6_f complex, and the ATP synthase. These multiportion complexes harness solar energy and, together with ATP synthase, produce reducing power (NADPH) and chemical energy (ATP) for the production of carbohydrates in the Calvin cycle [26–29].. The ubiquitination pathway is involved in nitrogen recycling and prevents senescence during herbicide stress [30]. In conclusion, the proteins encoded by the identified genes are involved in the metabolic detoxification, carbon metabolism, and repair of the PSII complex.

Understanding the genetics of herbicide tolerance in wheat will guide breeders in the development of herbicide-tolerant cultivars with wider safety margins. Metribuzin tolerance in wheat has high heritability and significant additive gene action with no epistasis. Therefore, MAS may be a feasible routine solution for selecting herbicide-tolerant lines in crop improvement programs. Metribuzin tolerance in wheat is most likely a non-target-based mechanism where metribuzin is detoxicated by a series of metabolic enzymes. However, transcriptome-wide gene expression profiling is needed to reveal genes and pathways endowing metabolic herbicide resistance in wheat.

## Conclusions

The simple additive-dominance mode of gene action suggests that a simple selection procedure could be successfully exploited in an early segregating generation to select lines for metribuzin tolerance breeding in wheat. The present investigation emphasized the degree of gene expression in the PSII assembly factor, antioxidants and detoxifying systems (CYPs, GSTs, GT, GPX) as the responsible factors for determining metribuzin tolerance in wheat. The identified markers could be used in marker-assisted selection of lines for breeding tolerant cultivars. Alternatively, tolerant genes could be introduced into elite wheat cultivars by natural introgression to enhance metribuzin tolerance.

## Methods

### Herbicide

Metribuzin (C_8_H_14_N_4_OS), a triazinone herbicide was purchased from Syngenta Crop Protection. Metribuzin binds its target site D1 protein in PSII and inhibits electron flow between the primary electron acceptor to plastoquinone, arresting photosynthesis. The metribuzin dose of 400 g a.i. ha^− 1^ was used to assess tolerance status in parents, F_1_, F_2_, BC_T_ and BC_S_ populations and F_5–7_ RILs of the cross, CM × R (for all abbreviations refer to Table 1).

### Plant material

**Fig. 4:**
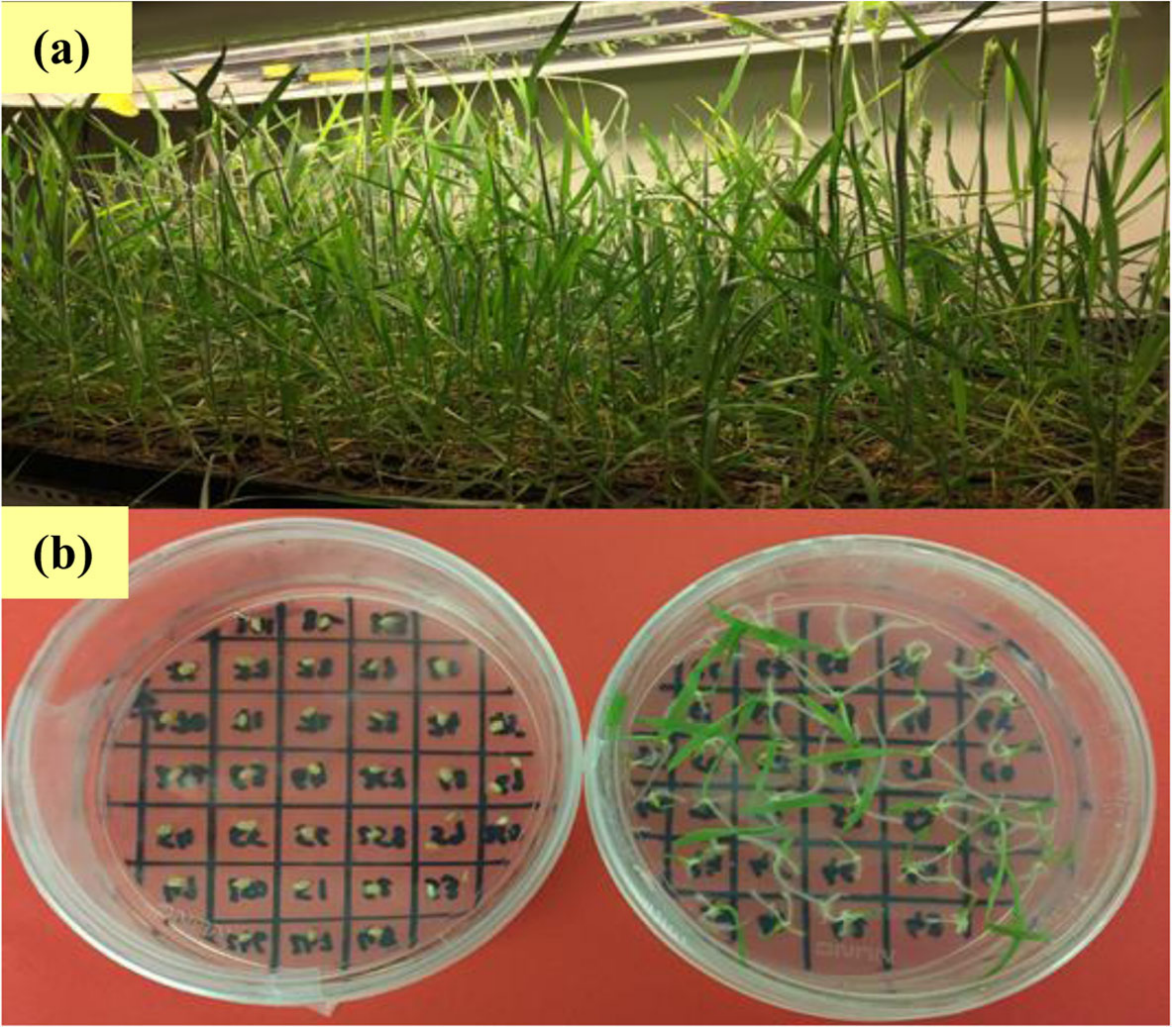
A Rapid generation single seed-descent method used to produce recombinant inbred lines of Chuan Mai 25 × Ritchie cross - (a) plants growing under a controlled environment room; and (b) wheat embryos (left) germinating in-vitro (right) in a culturing medium

Seven wheat genotypes with differential tolerance to metribuzin (Table 1) were obtained from Australian winter wheat collection. The tolerant and susceptible parents selected for this study were from previous tolerance screening [9] and local WA cultivars identified by Kleemann and Gill [31]. Plants of metribuzin T (tolerant) and S (susceptible) parental type were grown in 1 L pots containing potting mix (50% peat moss: 50% river sand) and maintained in a glasshouse at The University of Western Australia during a normal winter growing season. Single T and S plants growing individually in pots were paired according to floral synchronicity to produce F_1_ maternal R and paternal S (F_1_ RS) and F_1_ maternal S and paternal R (F_1_ SR) hybrids. Reciprocal crosses were used to check maternal effects of herbicide resistance. Subsequently, RS F_1_s were selfed and backcrossed to their R and S plants to produce F_2_ and backcross (BC_T_ and BC_S_) generations, respectively. Additionally, the Chuan Mai 25 (T) × Ritchie (S) cross was selected to develop recombinant inbred lines (RILs) in the growth chamber using rapid generation single seed-descent in-vitro embryo culture technique (Fig.4) [32]. A total of 73 F_5–7_ RILs were screened for metribuzin tolerance in the glasshouse to calculate heritability.

### Herbicide screening and phytotoxic assessment

The parents, F_1_, F_2_, BC_T_ and BC_S_ populations and F_5–7_ RILs of the cross, CM × R were evaluated for metribuzin tolerance in a sand-tray system [9]. The trays were sprayed with 400 g a.i. ha^− 1^ of metribuzin via a twin flat-fan nozzle, perpendicular to the tray surface in two passes at a flow rate of 118 L ha^− 1^ and 200 kPa pressure in a cabinet spray chamber. The amount of herbicide required for 400 g a.i. ha^− 1^ in L/ha was calculated using the ratio of herbicide rate by flowrate of twin flat-fan nozzle. The trays were maintained in a phytotron, where the temperature was set to 25/15 °C day/night and watered regularly every 48 h.

**Fig. 5:**
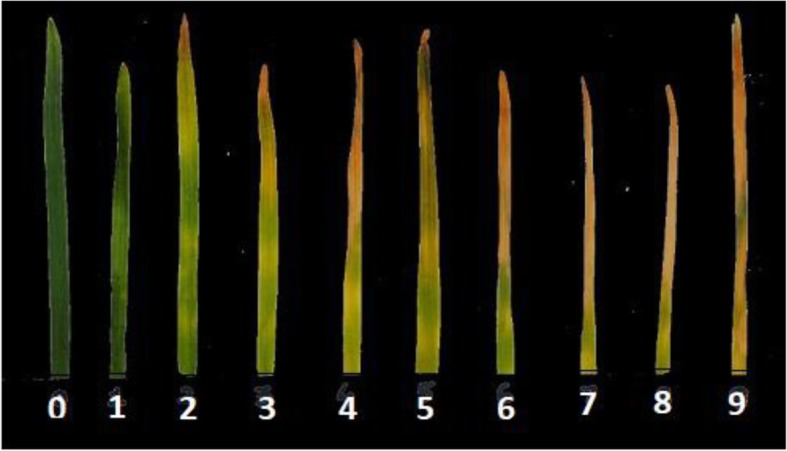
Leaf senescence rating from 0 to 9; plants with an average SS ≤ 3 recorded tolerant (T), 4 to 5 moderately tolerant (MT), and 6 to 9 as susceptible (S)

Senescence score (SS)/visual damage was measured 21 days after treatment (DAT) (Fig. 5). Plants with no visual symptoms were scored as 0, increasing levels of yellowing and stunting were scored from 1 to 4, increasing levels of leaf abnormalities (leaves wrinkling) and leaf necrosis were scored from 5 to 8, and dead plants with total leaf browning and necrosis of the apex were scored as 9. Lines with an average SS ≤ 3 recorded tolerant (T), 4 to 5 moderately tolerant (MT), and 6 to 9 as susceptible (S). For parents and F_1_ hybrids, SS was averaged over the three repeats.

### Identification of SNP and potential candidate genes

The distribution of alleles at the SNP sites was assessed using the wheat 90 K iSelect SNP genotyping assay, containing 81,587 genome-wide distributed SNPs following the procedure described by Wang et al. [33]. Allele calls were generated for the seven parents used in this study (Table 1), with the four tolerant genotypes as group 1 and three susceptible genotypes as group 2 for comparison. SNP clustering and genotype calling were performed using Genome Studio 2.0 software (Illumina). The monomorphic and poor-quality SNP markers, which had more than 20% missing values, ambiguous SNP calling, or minor allele frequencies below 5%, were excluded from further analyses. The polymorphic SNP loci between the two groups were used for candidate gene analysis.

The candidate genes controlling metribuzin tolerance were identified using BLASTN program, against the Ensembl Plants (*Triticum aestivum* IWGSC_refseqv1.0) to find the Traes numbers of genes. BLAST hits were filtered with an *e-*value threshold of 10^− 5^ and sequence similarity higher than 95%. The Traes numbers were searched in UniProt in TrEMBL (http://www.uniprot.org) and UniParc (https://www.uniprot.org/uniparc/) to obtain more information including protein domain, family, molecular and biological functions of the potential candidate genes. Further, the key features of the domain and InterPro annotation were searched in pfam and Prosite to check the characteristics of the protein. Only those genes with known function and/or related to photosynthesis and metabolic detoxification were considered as potential candidate genes for metribuzin tolerance in wheat.

### Principal component analysis (PCA)

PCA was performed on the SNP calls of the seven parents to determine genetic relatedness/diversity. SNP alleles were converted to a 1/0 binary system, followed by PCA performed using the built-in R function ‘prcomp’ and data was visualized using the ‘dudi.pca’ function from the ade4 R package [34] using SNP as variables.

### Genetic analyses

The contribution of maternal or cytoplasmic effects on the differences between population means was assessed by comparing the means of reciprocal F_1_ crosses. The mode of inheritance of metribuzin tolerance was estimated for each cross combination by generation mean analysis. Mean data on SS recorded on different generations, viz. parents (P_1_ and P_2_), F_1_, F_2_, BC_T_ and BC_S_ for nine cross combinations, were subjected to a scaling (A, B, C and D) and joint-scaling test using the weighted least squares method, which testifies the presence or absence of epistasis [35–37]. When the additive–dominance model fitted the data, a generation variance analysis was performed based on the method described by Allard [38]. This provided estimates of additive and dominance components of variance. The estimated gene effects: mean (m), additive (d) and dominance (d) values were tested by t-test at the 0.05 and 0.01 levels of probability. Further, the goodness-of-fit of the model was tested by comparing expected means of the six generations, calculated from the parameter estimates and observed generation means using chi-squared (χ^2^) statistic, and the significance of each parameter was tested using a *t*-test [35, 36].

The nature of dominance was determined from the potence ratio according to [38] P $=\frac{F_1-M.P.}{0.5\ \left({P}_2-{P}_1\right)}$, where P is the relative potence of the gene set, F_1_ is the first generation mean, P_1_ is the mean of the lower parent, P_2_ is the mean of the higher parent, and M.P. is the mid-parent value. Complete dominance was indicated when *P* was − 1 or + 1, while partial dominance was indicated when ‘*P*’ was − 1 or + 1, except for zero, which indicates the absence of dominance. Over dominance was indicated when the potence ratio exceeded + 1. The positive and negative signs indicate the direction of the dominance of either parent.

The generalized linear model based on Poisson regression was fitted to the SS data of F_5_, F_6_ and F_7_ RILs from the, Chuan Mai 25 × Ritchie cross using glm() function in R and heritability was calculated based on ANOVA using the formula: h^2^ = ${\delta}_g^2/$ (${\delta}_g^2+$ ${\delta}_e^2$) where ${\delta}_g^2$ and ${\delta}_e^2$ are the estimated genotypic and error variances, respectively. The estimated genotypic and error variances were calculated as: ${\delta}_g^2$ $=\frac{MSg- MSe}{r}$ and ${\delta}_e^2$ $=\frac{MSe}{r}$ where MSg is the mean square of the RILs, MSe is the residual error and r is the number of replicates. Further, the number of genes controlling metribuzin resistance in each cross was estimated using Wright’s formulae [39, 40].

## Supplementary information

**Additional file 1: Table S1.** The SNP’s and their alleles in tolerant and susceptible bulk.

## Data Availability

The datasets used and/or analyzed during the current study are available from the corresponding author on reasonable request.

## Abbreviations

BC_S_: Backcross of F1 to susceptible parent
BC_T_: Backcross of F1 to tolerant parent
CM: Chuan Mai 25
CYP: Cytochrome P450
d: Additive gene effect
D: Dagger
ER: Eagle Rock
F: Fundulea 490
F1: First filial generation
F2: Second filial generation
GPX: Glutathione peroxidase
GST: Glutathione S-transferase
GT: Glycosyltransferase
h: Dominance gene effect
K: Kite
MAS: Marker-assisted selection
mp: mid-parent value
PCA: Principlal component analysis
PSII: Photosystem II
R: Ritchie
S: Spear
SNP: Single nucleotide polymorphism
SS: Senescence score
